# Supplementary material for: Abruptly attenuated carbon sequestration with Weddell Sea dense waters by 2100
Source: Nat Commun. 2022 Jun 14;13:3402. doi: 10.1038/s41467-022-30671-3 (PMC9198026; doi:10.1038/s41467-022-30671-3)
Supplement: Supplementary file 1 — Supplementary Information [file 41467_2022_30671_MOESM1_ESM.pdf]

Abruptly attenuated carbon sequestration with Weddell Sea dense waters by 2100

Cara Nissen[1] Ralph Timmermann[1] Mario Hoppema[1] Özgür Gürses[1] Judith Hauck[1]

[1]Alfred Wegener Institut, Helmholtz Zentrum für Polar- und Meeresforschung, Bremerhaven, Germany

This supplementary material contains additional detail on the analysis, in support of the findings described in the main manuscript. In **Supplementary Section 1**, we describe the findings of the model extensions and in **Supplementary Section 2**, we discuss the dynamics which lead to the episodic acceleration of deep-ocean carbon accumulation via open-ocean mixing, which is only briefly summarized in the main text. Thereafter, additional figures are provided in **Supplementary Section 3**: Besides additional detail on the deep-ocean carbon budget, water mass properties, and water mass transformations in the southern and eastern Weddell Sea (Fig. S1-S12 & S19), an assessment of the model extensions (Fig. S13-S16), and a comparison of atmospheric air temperatures in the AWI Climate Model used to force the simulations in this study to other climate models contributing to the "Coupled Model Intercomparison Project Phase 6" (CMIP6; Fig. S20), Supplementary Figures S17 & S18 and S21 & S22 show the comparison of simulated fields of temperature, salinity, density, and dissolved inorganic carbon concentrations with available observations in the area of interest and the horizontal resolution of the mesh used for the FESOM-REcoM simulations in this study. The following Figures are included in this document:

Fig. S1 Upper-ocean carbon inventory in the southern Weddell Sea over the 21st century.

Fig. S2: Components contributing to the biologically-driven deep-ocean carbon accumulation.

Fig. S3: Distribution of total carbon concentrations in the southern Weddell Sea.

Fig. S4: Vertical carbon mixing in the deep ocean of the southern Weddell Sea.

Fig. S5: Vertical carbon advection in the deep ocean of the southern Weddell Sea.

Fig. S6: Vertical volume transport in the southern Weddell Sea.

Fig. S7: Bottom water ventilation and density distribution in the 2080s.

Fig. S8: Deep convection in the southern Weddell Sea.

Fig. S9: Property changes of Warm Deep Water in the southern Weddell Sea.

Fig. S10: Water mass transformations due to evaporation minus precipitation and heat fluxes.

Fig. S11: Water mass properties on the eastern Weddell Sea shelf.

Fig. S12: Water mass transformations due to buoyancy fluxes on the eastern Weddell Sea shelf.

Fig. S13: Deep-ocean carbon accumulation in the model extensions.

Fig. S14: Evolution of air temperatures and bottom density in the model extensions.

Fig. S15: Divergence of physical flux components in the model extensions and vertical diffusivity.

Fig. S16: Changes in sea ice growth, basal melt, and water mass properties in the model extensions.

Fig. S17: Model evaluation of density in the southern Weddell Sea.

Fig. S18: Model evaluation of temperature and salinity in the southern Weddell Sea.

Fig. S19: Southern Weddell Sea carbon budget below 2500 m and 3000 m.

Fig. S20: Air temperatures in the AWI Climate Model in comparison to other climate models.

Fig. S21: Grid resolution in the Weddell Sea sector of the global FESOM-REcoM simulations in this study.

Fig. S22: Model evaluation of dissolved inorganic carbon in the southern Weddell Sea.

1 Model extensions

As described in more detail in the Method section, atmospheric CO_2 levels continue to rise throughout both model extensions (*ext1* and *ext2*), whereas both extensions were run under the assumption that the atmospheric temperature stabilizes (at two different constant near-end-of-century levels; Fig. S14). As a consequence of the rise in atmospheric CO_2 levels, the oceanic uptake of CO_2 in the southern Weddell Sea increases to 85 Tg C yr^{-1} by the end of the model extensions (Fig. S1a), and the upper ocean carbon inventory continues to rise accordingly (Fig. S1b). At the same time, following the decade of lowest deep-ocean carbon accumulation in the southern Weddell Sea in *simA*, carbon accumulation stays low for the first two decades in *ext1*, before another high-accumulation phase occurs (thick dark grey line in Fig. S13). In *ext2*, which is forced with lower atmospheric temperatures than *ext1* (Fig. S14a) , these high-accumulation events are more frequent (thin dark grey line in Fig. S13), leading to almost twice as much additional accumulation after 50 years in *ext2* (0.58 Pg C) than in *ext1* (0.32 Pg C). In agreement with *simA*, the variability of carbon accumulation rates in the deep ocean of the southern Weddell Sea in both extensions are mainly controlled by physical fluxes (blue line in Fig. S13a), with open-ocean mixing being the flux component controlling the high-accumulation events (Fig. S15). In fact, without open-ocean mixing, total deep-ocean carbon accumulation between 2080 and the end of the model extensions would be 52% and 55% lower for *ext1* and *ext2*, respectively (light grey lines in Fig. S13). Taking *ext1* as an example, also the residual average carbon accumulation rate between 2080 and year 50 of this extension (3.2 Tg C yr^{-1}) can largely be explained by biological fluxes (88%), implying that the low deep-ocean carbon transfer with Weddell Sea dense waters towards the end of *simA* is sustained also in this model extension at stabilized near-end-of-century atmospheric temperatures.

Towards the end of the 21st century in *simA*, the lower density of newly formed dense waters on the continental shelves prevents these from reaching the abyss in the open-ocean Weddell Sea. Overall, the simulated changes in water mass properties on the southern continental shelves are sustained throughout the model extensions (especially for *ext1*, see Fig. S16 & Fig. S16). In *ext1*, sea ice formation stabilizes at a rate close to that projected for the 2090s (Fig. S16a; possibly a consequence of the constant atmospheric forcing applied in the extension), whereas basal melting has further increased to 120% of that in the 1990s by the end of the extension (Fig. S16b; possibly in response to the further increase in the heat content of waters over the shelf, see Fig. S16f). As a consequence, the decoupling between bottom waters on the southern continental shelves and those in the open ocean is sustained in both model extensions. After 2100, the density difference stabilizes at 0.37 kg m^{-3} in *ext1* and 0.33 kg m^{-3} in *ext2*. While this is lower than the density difference in the 2090s (0.44 kg m^{-3}), a stabilization is expected due the setup of the model extensions (no further warming after 2100; Fig. S14a) and should therefore not be interpreted as a reversal of the simulated decoupling between the continental shelf sea and the deep ocean over the 21st century (see Methods). More importantly, given that the density difference is still at least three times larger by the end of the model extensions than in the 1990s, the sustained reduced connectivity implies that more than a few decades and possibly also a larger reversal of the end-of-century warming than applied in *ext2* are needed to re-establish the pathway for newly formed dense waters from the continental shelf sea to the abyss of the southern Weddell Sea.

2 Episodic enhancement in deep-ocean carbon accumulation rates

Compared to the preceding and subsequent decades, the downward transfer of carbon is enhanced in the southern Weddell Sea in the 2080s, the 2120s of *ext1* and regularly in *ext2* (see Fig. 2a in the main text). Here, we will exemplarily discuss the dynamics leading to the accelerated accumulation in the 2080s. Since neither oceanic CO_2 uptake nor biological sinking fluxes are enhanced in this decade (Fig. 2a & b in the main text) and thereby fail to explain the increased downward carbon flux, this signal is driven by an enhanced downward physical transport. In fact, as shown in Fig. 3a of the main manuscript, vertical mixing in the southern Weddell Sea amounts to $\sim$11 Tg C yr^{-1} in the 2080s (with 70% of that taking place in the open ocean, see also the elevated vertical diffusivity in the open ocean in Fig. S4d), largely explaining the increased downward transfer of carbon in this decade. In general, this high mixing flux could be either due to changes within the southern Weddell Sea or due to changes in the properties of source waters imported on the eastern flank of the Weddell Gyre (or a combination of both). However, given that the decline both in the ventilation of bottom waters and in the density of waters at different depth levels along the southwestern continental slope is similar in the 2080s to that in the 2090s (relative to the 1990s; compare Fig. S7 to Fig. 4 in the main text), the transfer of dense waters from the southern continental shelf sea to the abyss is not enhanced in the 2080s, implying that the enhanced downward mixing in the 2080s is mostly driven by changes upstream.

In fact, in the 2080s, bottom water ventilation is enhanced on the eastern side of the southern Weddell Sea (Fig. S7a & also Fig. S6). This positive ventilation anomaly extends from the sea floor all the way to a water depth of ∼1km at the transect "WS east" (Fig. S7a & c), suggesting that these waters have relatively recently been in touch with the upper ocean, where they have possibly been enriched in carbon (see Fig. 3d in the main text and Fig. S3). Further, since these waters close to the eastern Weddell Sea continental slope have a high-density signature (much higher than the high-density signature due to enhanced upwelling of waters from below in the more central parts of the southern Weddell Sea; see Fig. S7e & f and compare to Fig. 4 in the main text), it seems plausible that these waters - after their inflow on the eastern side - are mixed to below 2000 m in the open ocean south of the transect SR4, leading to the largest signature of enhanced deep-ocean carbon accumulation in this area.

3 Additional figures

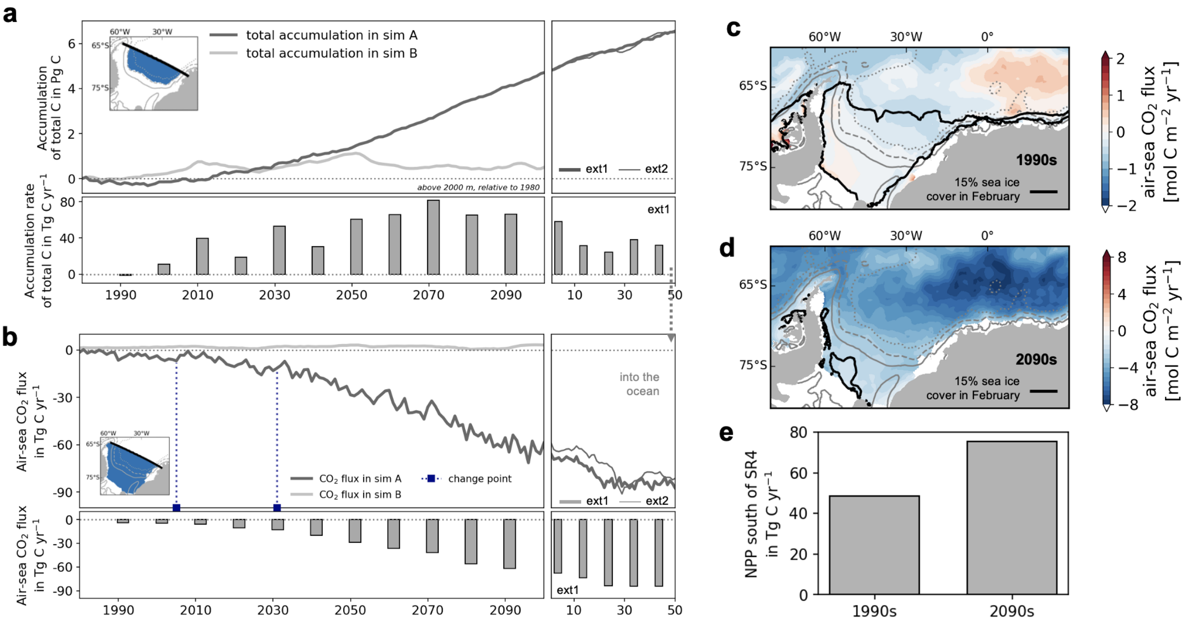

Figure S1: Upper-ocean carbon inventory over the 21st century. **a** Accumulated carbon in Pg C above 2000 m and **b** annually integrated air-sea CO_2 flux in Tg C yr^{-1} in the southern Weddell Sea south of the transect SR4 of the World Ocean Circulation Experiment (see blue areas in inlets) in the model simulation *simA* (dark grey; historical + SSP5-8.5 scenario) and the control simulation *simB* (light grey). By the year 2100, the carbon inventory in the top 2000 m has increased by 4.71 Pg C in this region, while the deep-ocean inventory has increased by 0.75 Pg C (see Fig. 2 in the main text). In panels **a,b**, bars depict the average **a** carbon accumulation rates and **b** air-sea CO_2 flux for each decade. After the year 2100, thick and thin dark grey lines correspond to the extensions *ext1* and *ext2* and bars show the results for *ext1*. Note that due to their setup, the time axis of the model extensions corresponds to simulation years rather than calendar years. See Method section of the main manuscript for details. In panel **b**, change points in the time series are indicated with vertical lines and as squares on the x axis (see Methods and references[1,2]). **c,d** Air-sea CO_2 flux in mol m^{-2} yr^{-1} in the Weddell Sea in *simA* averaged over the **c** 1990s and **d** 2090s. Isolines of 15% sea-ice cover in February in the respective decade are indicated by the black contours. Grey contours show the 700 m (solid), 2000 m (dashed), and 3500 m (dotted) isobaths. For panels **b-d**, a negative CO_2 flux denotes a flux into the ocean. **e** Vertically integrated annual net primary production (NPP) in Tg C yr^{-1} south of the transect SR4 (see inlet in panel **b**) in *simA* averaged over the 1990s and the 2090s.

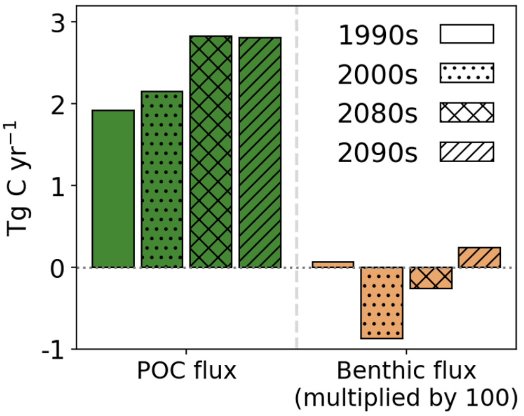

Figure S2: Components contributing to the biologically-driven deep-ocean carbon accumulation. Average annual accumulation rate of carbon in Tg C yr $^{-1}$ south of the transect SR4 of the World Ocean Circulation Experiment and below 2000 m in *simA* (historical + SSP5-8.5 scenario; see Methods in the main text) due to sinking fluxes of particulate organic carbon (POC; green) and fluxes of dissolved inorganic carbon from the benthic layer (brown). Benthic fluxes are multiplied by 100 to fit the scale, demonstrating the dominance of POC fluxes for the accumulation of carbon in the deep ocean due to biological fluxes. Rates are given for the 1990s, 2000s, 2080s, and 2090s, as indicated by the hatching of the bars.

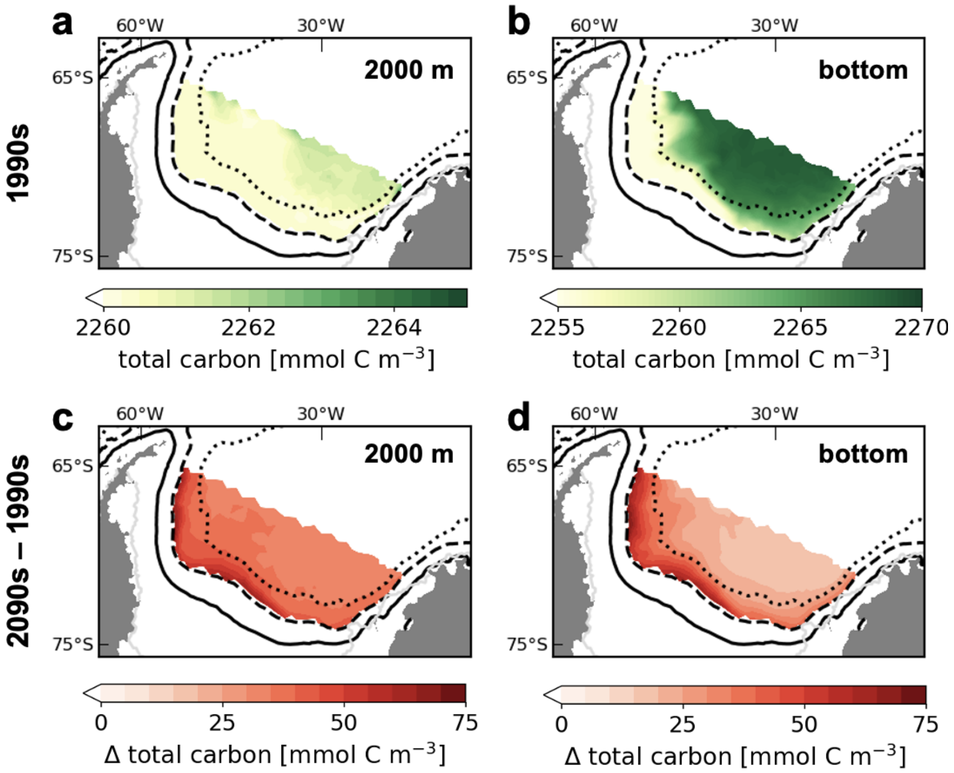

Figure S3: Distribution of total carbon concentrations in the southern Weddell Sea. **a, b** Average total carbon concentrations in mmol C m^{-3} in the 1990s of *simA* (historical + SSP5-8.5 scenario; see Methods in the main text) at **a** 2000 m and **b** at the bottom. Note the different scales. **c, d** Same as panels **a, b**, but for the difference between the 2090s and the 1990s.

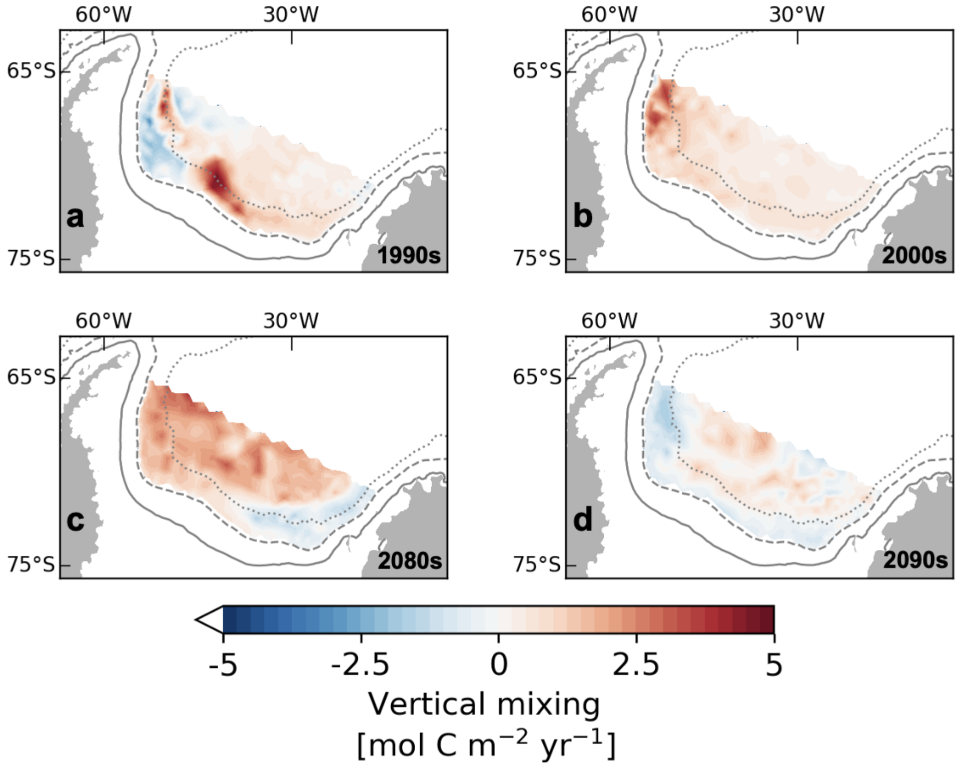

Figure S4: Vertical carbon mixing in the deep ocean of the southern Weddell Sea. The divergence of vertical mixing of carbon in mol C m^{-2} yr^{-1} in the southern Weddell Sea south of transect SR4 of the World Ocean Circulation Experiment and below 2000 m in the **a** 1990s, **b** 2000s, **c** 2080s, and **d** 2090s in *simA* (historical + SSP5-8.5 scenario; see Methods in the main text). Positive fluxes denote an increase in the deep-ocean carbon inventory in the volume of interest due to this flux component. Grey contours show the 700 m (solid), 2000 m (dashed), and 3500 m (dotted) isobaths.

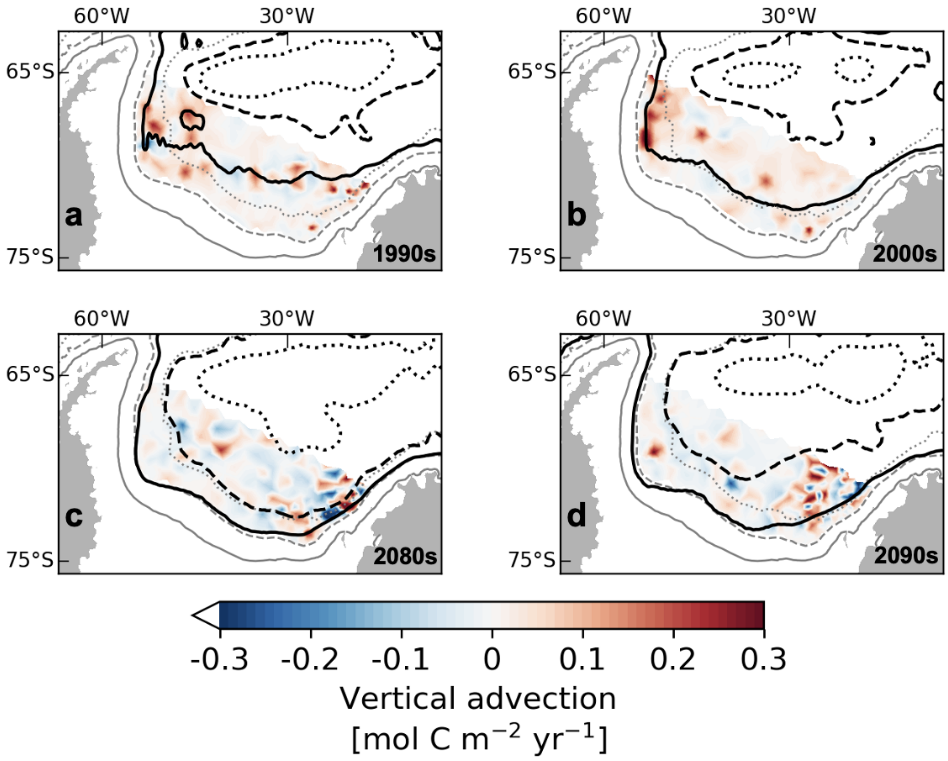

Figure S5: Vertical carbon advection in the deep ocean of the southern Weddell Sea. The divergence of vertical advection of carbon in mol C m^{-2} yr^{-1} in the southern Weddell Sea south of transect SR4 of the World Ocean Circulation Experiment and below 2000 m in the **a** 1990s, **b** 2000s, **c** 2080s, and **d** 2090s in *simA* (historical + SSP5-8.5 scenario; see Methods in the main text). Positive fluxes denote an increase in the deep-ocean carbon inventory in the volume of interest due to this flux component. Grey contours show the 700 m (solid), 2000 m (dashed), and 3500 m (dotted) isobaths. Black contours denote selected isolines of the barotropic stream function in the respective decades, namely 5 Sv (solid), 12 Sv (dashed), and 20 Sv (dotted), indicating an intensification of the gyre circulation in the southern Weddell Sea over the 21stcentury in *simA*.

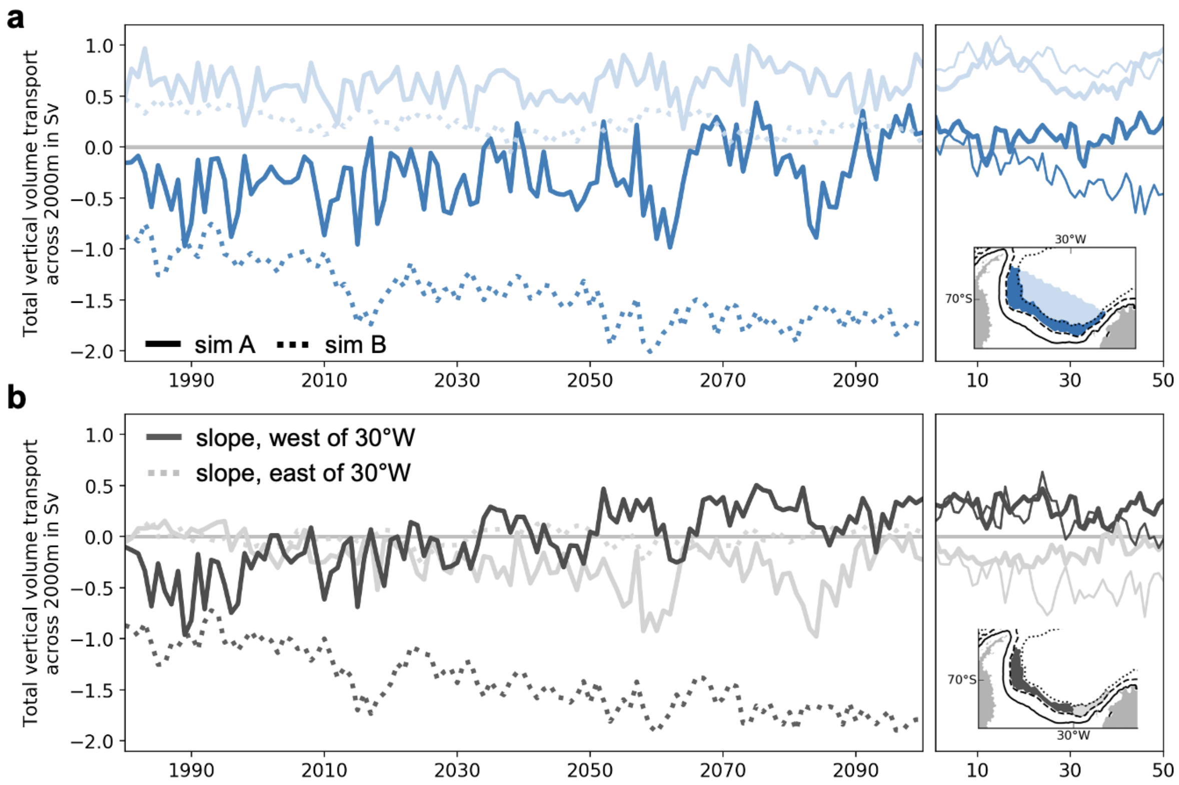

Figure S6: Vertical volume transport in the southern Weddell Sea. Vertical volume transport across 2000 m in Sverdrup (Sv; 1 Sv is $1{\cdot}10^6$ m^3 s^{-1}) south of the transect SR4 of the World Ocean Circulation Experiment in the model simulation *simA* (solid lines; historical + SSP5-8.5 scenario) and the control simulation *simB* (dotted lines). Fluxes are integrated over **a** the continental slope (dark blue) and the open ocean (light blue), which are separated at the 3500 m isobath (see inlet), and the continental slope **b** west (black) and east (light grey) of 30°W (see inlet). After the year 2100, thick and thin lines correspond to the extensions *ext1* and *ext2*, respectively. Note that due to their setup, the time axis of the model extensions corresponds to simulation years rather than calendar years. See Method section of the main manuscript for details.

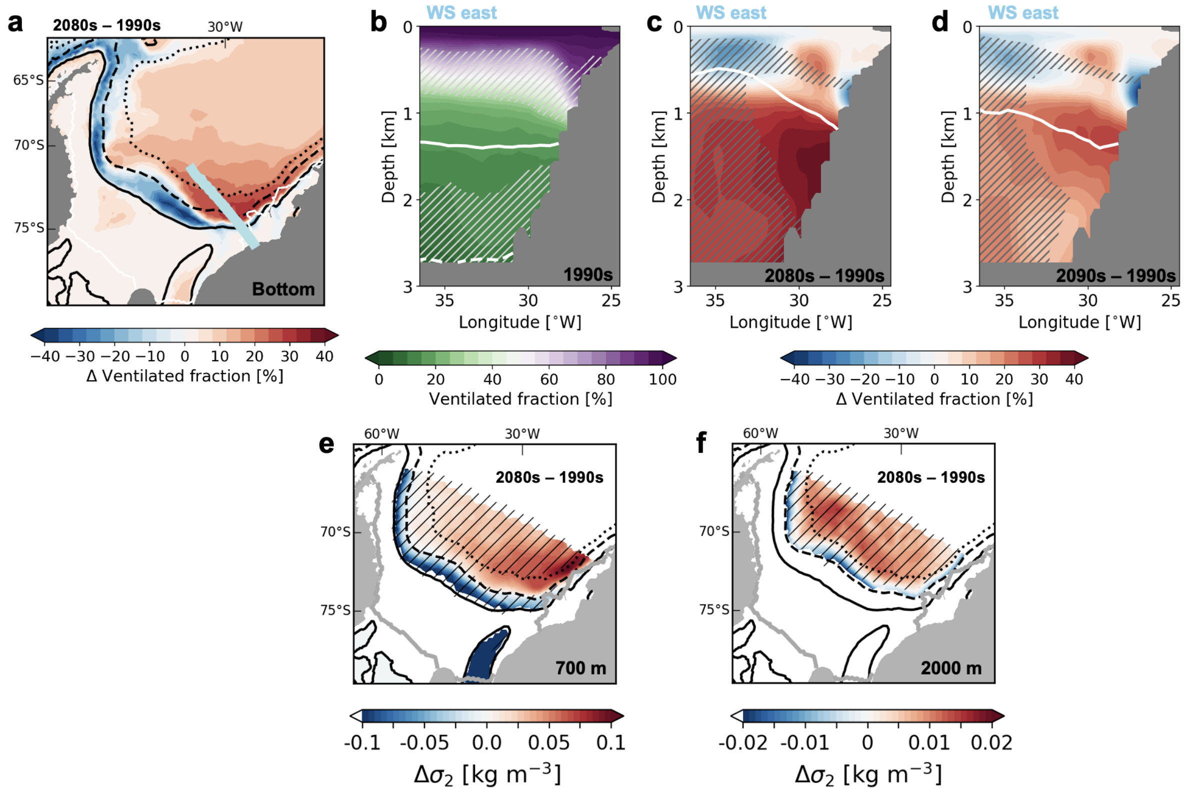

Figure S7: Bottom water ventilation and density distribution in the 2080s. **a** Difference in the age-tracer-based fraction of water that is ventilated at the bottom between the 2080s and the 1990s. The position of a transect in the southeastern Weddell Sea (WS east) is indicated in blue. **b-d** Age-tracer-based fraction of water that is ventilated at WS east in **b** the 1990s, **c** in the 2080s relative to the 1990s, and **d** in the 2090s relative to the 1990s. **e & f** Change in the distribution of potential density anomalies (σ_2 in kg m^{-3}, i.e., potential density referenced to 2000 dbar minus 1000 kg m^{-3}) between the 2080s and the 1990s at **e** 700 m and **f** 2000 m. In the transects in panels **b-d**, σ_2 isolines for the **b** 1990s, **c** 2080s, and **d** 2090s are shown as the solid (37.2 kg m^{-3}) and dotted (37.25 kg m^{-3}) white contour, respectively. In the maps in panels **e & f**, black contours show the 700 m (solid), 2000 m (dashed), and 3500 m (dotted) isobaths. In panels **b-f**, hatching denotes the presence of Weddell Sea Deep Water and Weddell Sea Bottom Water in the **a,b** 1990s and **c,d** 2090s, defined in the model as waters with a surface-referenced potential temperature $<$-0.2°C and a practical salinity$>$34.55.

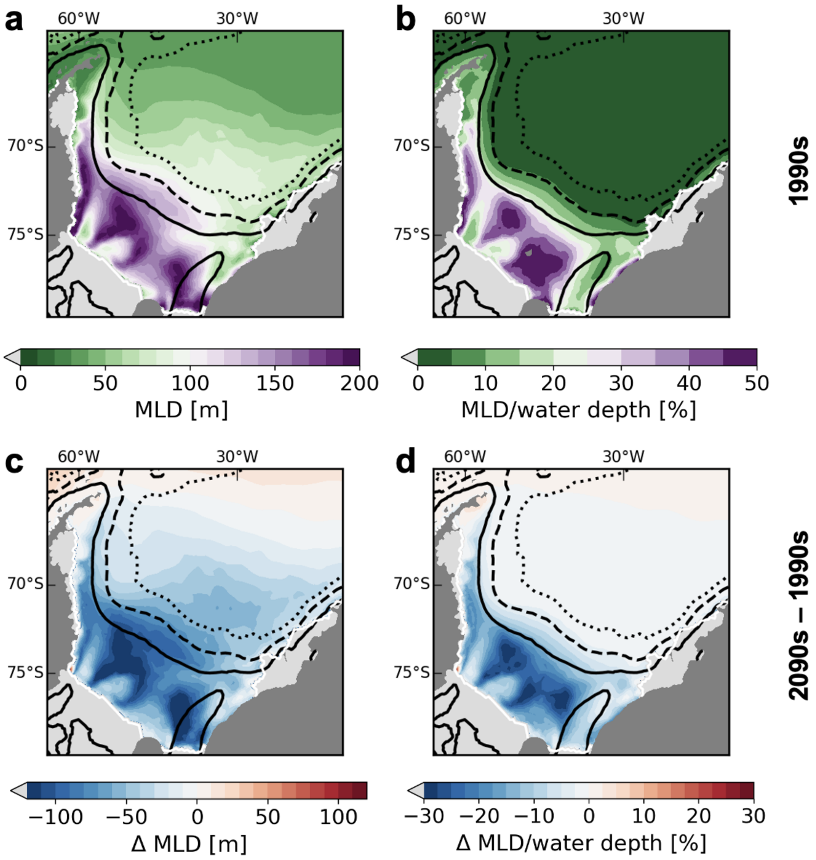

Figure S8: Deep convection in the southern Weddell Sea. **a** Annual mean mixed layer depth (MLD; m) in the southern Weddell Sea in the 1990s in *simA* (historical + SSP5-8.5 scenario; see Methods in the main text). The MLD is determined with the 0.125 kg m^{-3} density criterion[3] . **b** Same as panel **a**, but given in % relative to the water depth. This metric can be interpreted as an indicator of deep convection, with open-ocean deep convection typically being defined as areas where this quantity exceeds 50%[3] . For the annual mean MLD in *simA*, this threshold is not exceeded anywhere in our focus region. **c & d** Same as panels a & b, but for the difference between the 2090s and the 1990s. Black contours in all panels show the 700 m (solid), 2000 m (dashed), and 3500 m (dotted) isobaths.

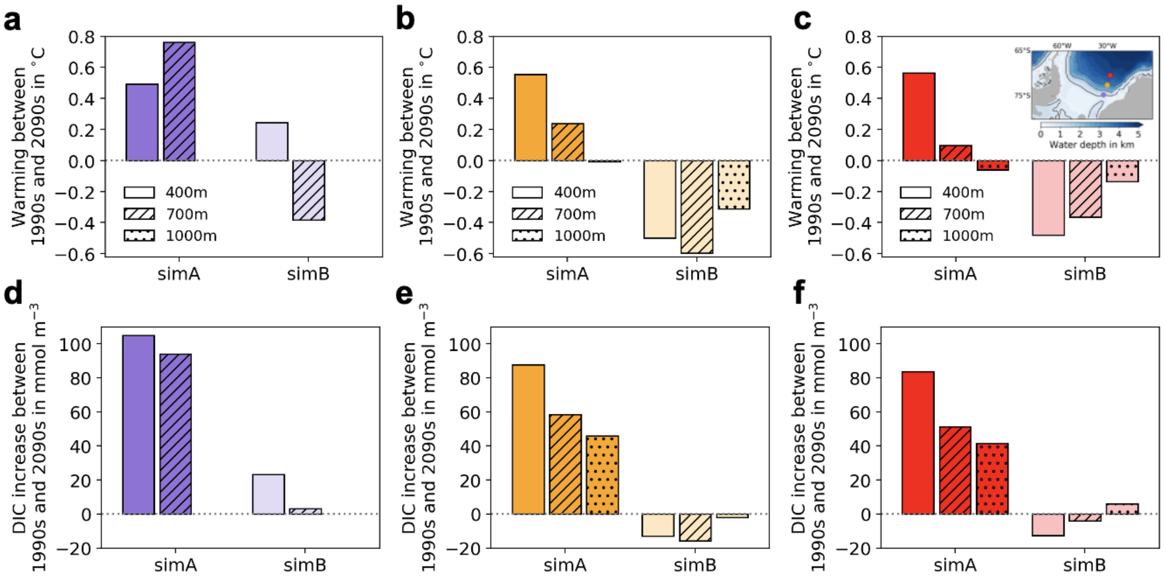

Figure S9: Property changes of Warm Deep Water in the southern Weddell Sea. **a-c** Temperature difference in °C between the 2090s and 1990s at three locations and at three depths (400 m, 700 m, and 1000 m) of the Warm Deep Water (WDW) core in the southern Weddell Sea as indicated by the different colors and the different hatching of the bars, respectively. See also Fig. S17. The temperature differences are shown for *simA* (darker colors) and *simB* (lighter colors). The locations are marked in the same colors on the map in panel **c**. Black contours in the map show the 700 m (solid), 2000 m (dashed), and 3500 m (dotted) isobaths. **d-f** Same as panels **a-c**, but for the change in dissolved inorganic carbon (DIC) concentrations in mmol m^{-3}.

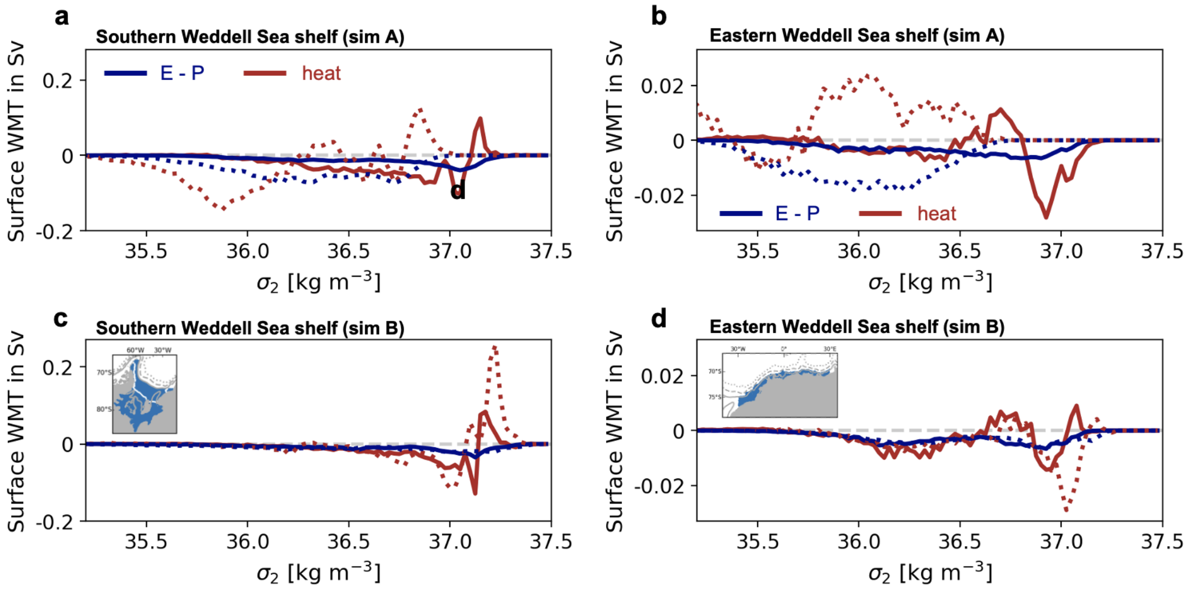

Figure S10: Water mass transformations due to evaporation minus precipitation and heat fluxes on the Weddell Sea continental shelf. **a & b** Surface water mass transformation rates (WMT) in Sverdrup (Sv; 1 Sv is $1{\cdot}10^6$ m^3 s^{-1}) as a function of the potential density anomaly (σ_2 in kg m^{-3}, i.e., potential density referenced to 2000 dbar minus 1000 kg m^{-3}) due to evaporation minus precipitation (E-P; blue) and due to heat fluxes (red) on the **a** southern and **b** eastern Weddell Sea continental shelf (see blue area in maps) in the 1990s (solid) and 2090s (dotted) from *simA* (historical + SSP5-8.5 scenario, see Methods in the main text). **c & d** denote the same quantities as panels **a & b**, respectively, but from the control simulation (*simB*). Positive transformations denote a densification of surface waters due to the buoyancy fluxes. Note that transformations due to freshwater fluxes from evaporation minus precipitation and heat fluxes are generally an order of magnitude smaller than those from sea ice and ice shelves (see Fig. S12 and Fig. 7 in the main text).

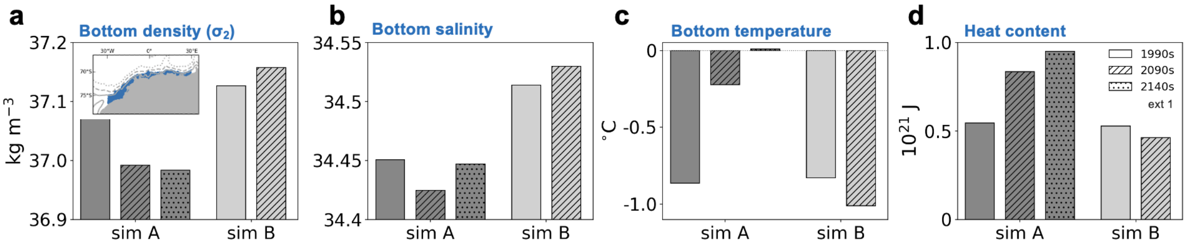

Figure S11: Water mass properties on the eastern Weddell Sea continental shelf. **a** Bottom potential density anomaly (σ_2 in kg m^{-3}, i.e., potential density referenced to 2000 dbar minus 1000 kg m^{-3}) on the eastern Weddell Sea shelf (blue area in the map) in the 1990s (plain bar), 2090s (hatched bar), and 2140s (dotted bar) in the experiment *simA+ext1* (dark grey; historical + SSP5-8.5 scenario + extension 1; see Methods in main text) and in the control simulation *simB* (light grey). **b-d** Same as panel **a,** but for **b** bottom salinity, **c** bottom potential temperature in °C, and **d** total heat content in 10^{21} J.

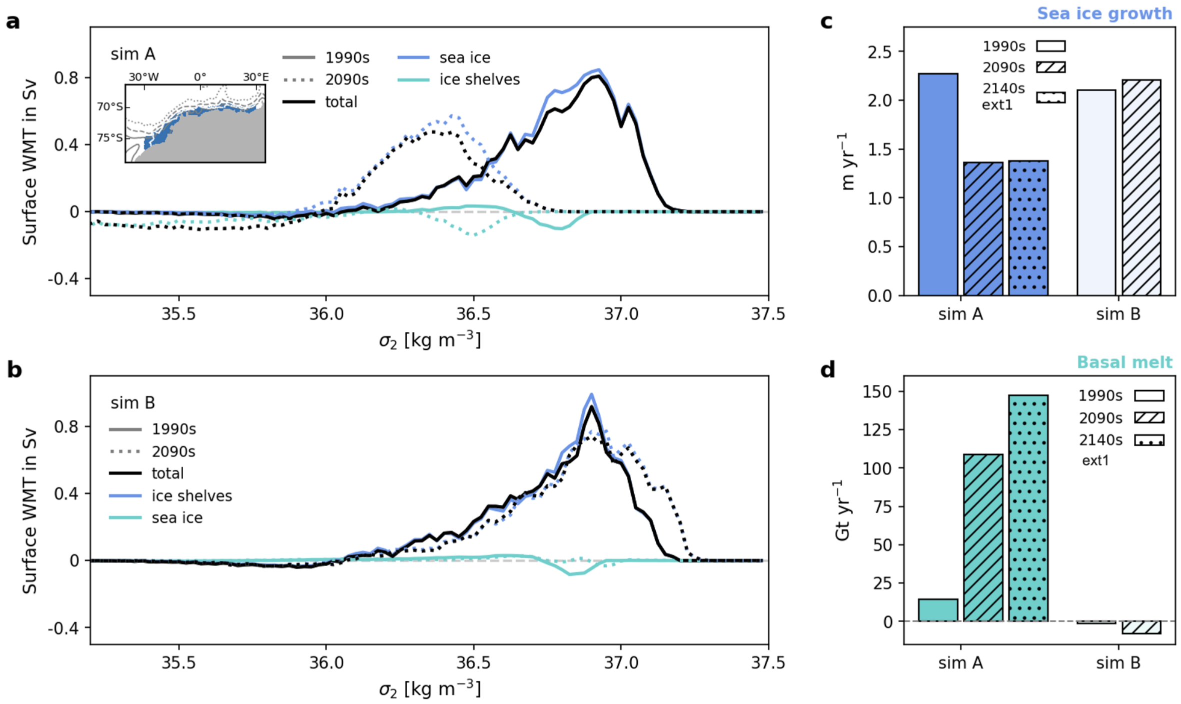

Figure S12: Water mass transformations due to buoyancy fluxes on the eastern Weddell Sea shelf. **a, b** Surface water mass transformation rates (WMT) in Sverdrup (Sv; 1 Sv is $1{\cdot}10^6$ m^3 s^{-1}) as a function of the potential density anomaly (σ_2 in kg m^{-3}, i.e., potential density referenced to 2000 dbar minus 1000 kg m^{-3}) due to the total buoyancy fluxes (black) and due to buoyancy fluxes from sea ice (blue) and ice shelves (mint) on the eastern Weddell Sea continental shelf (see blue area in map) in the 1990s (solid) and 2090s (dotted) from **a** *simA* (historical + SSP5-8.5 scenario, see Methods in the main text) and **b** the control simulation *simB*. Positive transformations denote a densification of surface waters due to buoyancy fluxes. Transformations due to heat fluxes and freshwater fluxes from evaporation minus precipitation are an order of magnitude smaller than those shown here (see Fig. S10). **c** Sea-ice growth in m yr^{-1} and **d** ice-shelf basal melt rates in Gt yr^{-1}. The bars in panels **c & d** denote averages for the 1990s (plain), the 2090s (hatched), and the 2140s (dotted) in *simA+ext1* (darker colors) and *simB* (lighter colors), respectively.

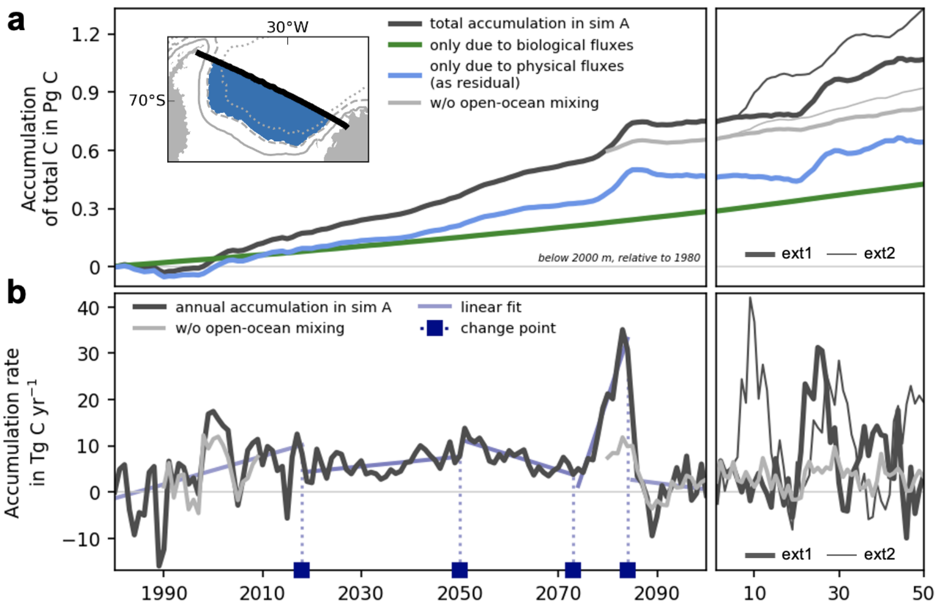

Figure S13: Deep-ocean carbon accumulation in the model extensions. **a** Accumulated carbon in Pg C between 1980 and 2100 below 2000 m in the southern Weddell Sea south of the transect SR4 of the World Ocean Circulation Experiment (see blue area in inlet) in the model simulation *simA* (dark grey; historical + SSP5-8.5 scenario) and for the 50 years of the model extensions *ext1* (thick dark grey) and *ext2* (thin dark grey). The contribution of biological and physical fluxes (the latter calculated as residual) of *simA*+*ext1* is shown in green and blue, respectively. **b** Annual carbon accumulation rates in Tg C yr^{-1} for *simA* (dark grey) and the model extensions *ext1* (thick dark grey) and *ext2* (thin dark grey). As in Fig. 2 of the main manuscript, the dark blue lines indicate the statistical models providing the best fits to describe the time series, and change points are indicated with vertical lines and as squares on the x axis (see Methods and references[1,2]). The light grey lines in both panels denote the accumulated carbon due to all processes except vertical mixing in the open ocean north of the 3500 m isobath (dotted line in the map in panel **a**). Note that due to their setup, the time axis of the model extensions corresponds to simulation years rather than calendar years. See Method section of the main manuscript for details.

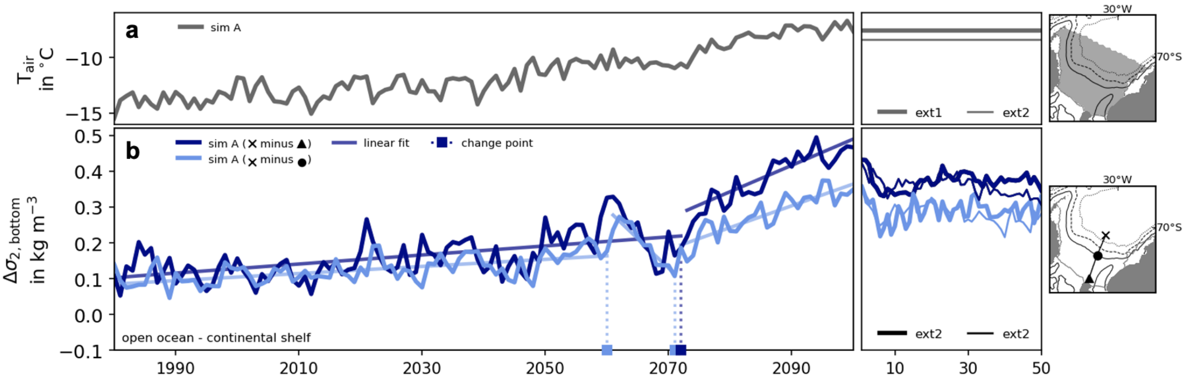

Figure S14: Evolution of air temperatures and bottom density in the model extensions. Panel **a** shows the average air temperature in °C over the southern Weddell Sea (see map) used to force *simA* between 1980 and 2100 (historical + SSP5-8.5). After the year 2100, the model experiment is extended for another 50 years by forcing it with constant air temperatures as *ext1* (thick line) and *ext2* (thin line), respectively. Panel **b** shows the difference in bottom potential density anomaly (σ_2 in kg m^{-3}, i.e., potential density referenced to 2000 dbar minus 1000 kg m^{-3}) between the open ocean and two locations on the continental shelf (light and dark blue, respectively) between 1980 and 2100 in *simA* and in the model extensions *ext1* (thick lines) and *ext2* (thin lines). See symbols in the map for the exact locations used to compute the density differences. The straight lines indicate the statistical models providing the best fits to describe the time series, and change points are indicated with vertical lines and as squares on the x axis (see Methods and references[1,2]). Note that due to their setup, the time axis of the model extensions corresponds to simulation years rather than calendar years. See Method section of the main manuscript for details.

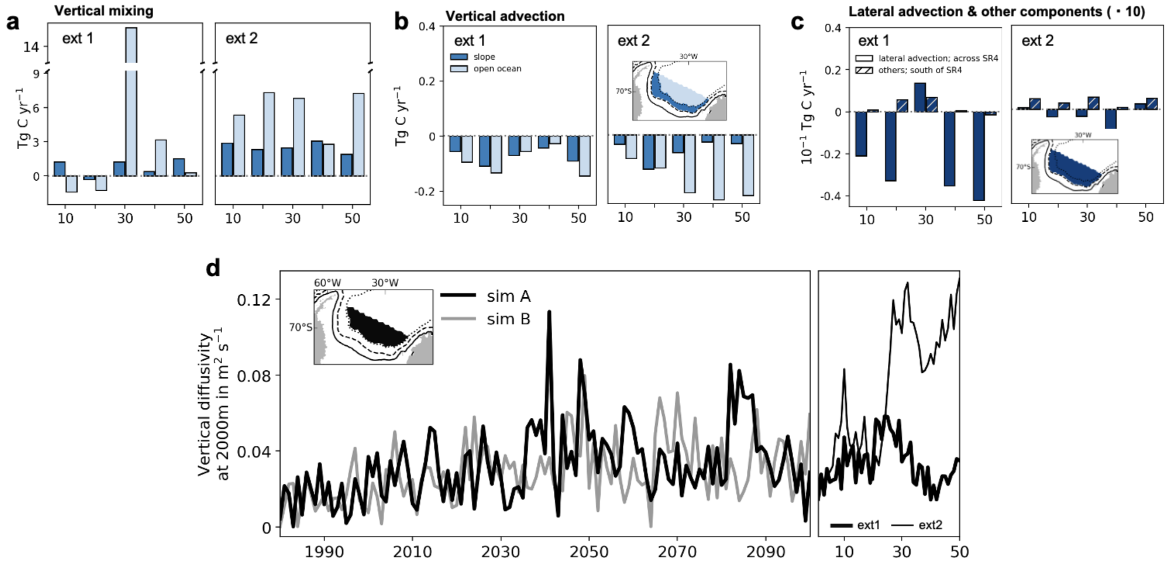

Figure S15: Divergence of physical flux components in the model extensions and vertical diffusivity in the southern Weddell Sea. Changes in the total carbon inventory south of transect SR4 of the World Ocean Circulation Experiment and below 2000 m in the model extensions *ext1* and *ext2* due to **a** vertical mixing across 2000 m, **b** vertical advection across 2000 m, and **c** lateral advection across the transect SR4 and the sum of all other flux components, e.g., from the eddy parametrization (dashed bars). All fluxes are in Tg C yr^{-1}, but note that the fluxes in panel **c** are one order of magnitude smaller than those in panels **a, b**. Positive fluxes denote an increase in the deep-ocean carbon inventory in the volume of interest due to the respective flux component. Panel **d** shows the average vertical diffusivity in m^2 s^{-1} at 2000 m in the open ocean (see inlet) in *simA* (black; historical + SSP5-8.5 scenario) and the control simulation *simB* (grey). After the year 2100, thick and thin lines correspond to the model extensions *ext1* and *ext2*, respectively. Note that due to their setup, the time axis of the model extensions corresponds to simulation years rather than calendar years. See Method section of the main manuscript for details.

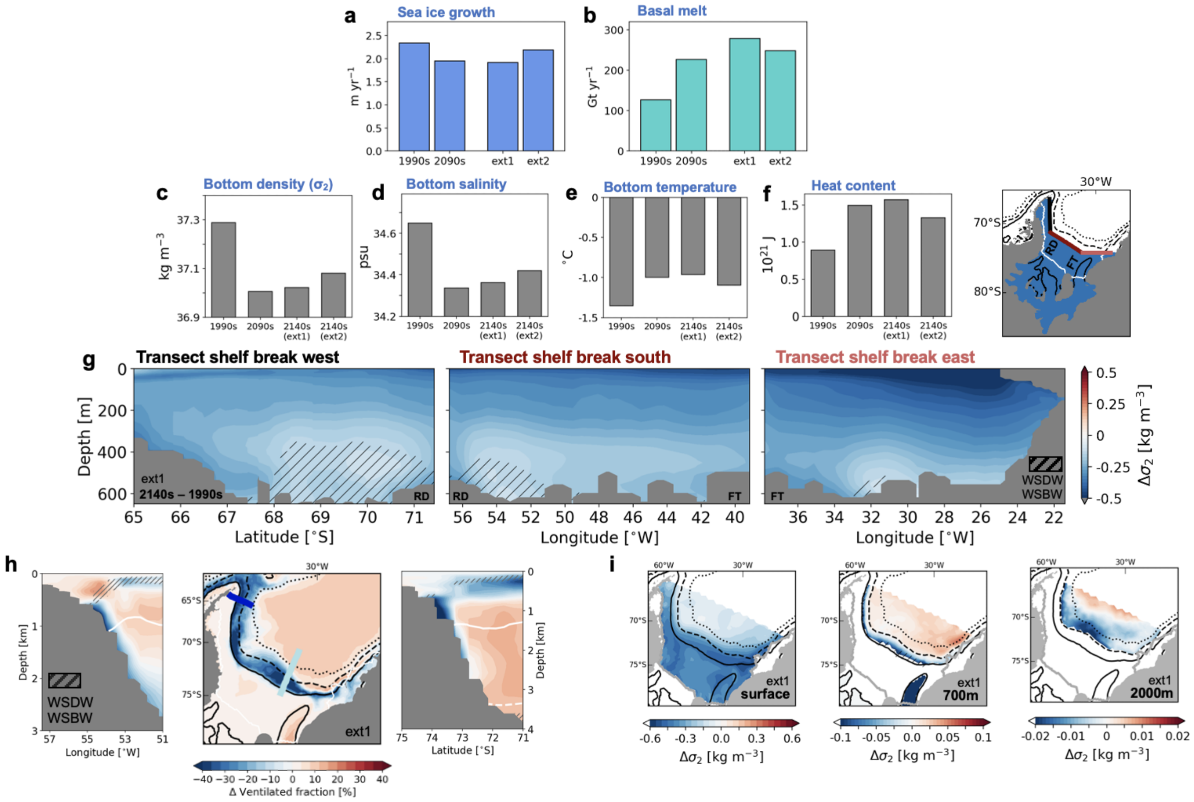

Figure S16: Changes in sea ice growth, basal melt, and water mass properties in the model extensions. **a** Sea-ice growth in m yr^{-1}, **b** ice-shelf basal melt rates in Gt yr^{-1}, **c** bottom potential density anomaly (σ_2 in kg m^{-3}, i.e., potential density referenced to 2000 dbar minus 1000 kg m^{-3}), **d** bottom salinity, **e** bottom potential temperature in °C, and **f** total heat content in 10^{21} J in the 1990s and 2090s in *simA* and the final decade of the model extensions *ext1* and *ext2*. All quantities are averaged for the southern continental shelf (blue area in the map). **g** Change in the distribution of σ_2 at a transect along the continental shelf break (see map) between the last decade in *ext1* and the 1990s in *simA*. Panels **h, i** show the change between the last decade in *ext1* and the 1990s in *simA* in **h** the age-tracer-based fraction of water that is ventilated at the bottom (middle) and across the continental slope in the western (SR4 west; left) and southern Weddell Sea (WS south; right) and **i** the distribution of potential density anomalies (σ_2 in kg m^{-3}) at the surface, 700 m, and 2000 m. Hatching in panels **g-i** denotes the presence of Weddell Sea Deep Water and Weddell Sea Bottom Water in the last decade of *ext1*, defined in the model as waters with a surface-referenced potential temperature $<$-0.2°C and a practical salinity$>$34.55.

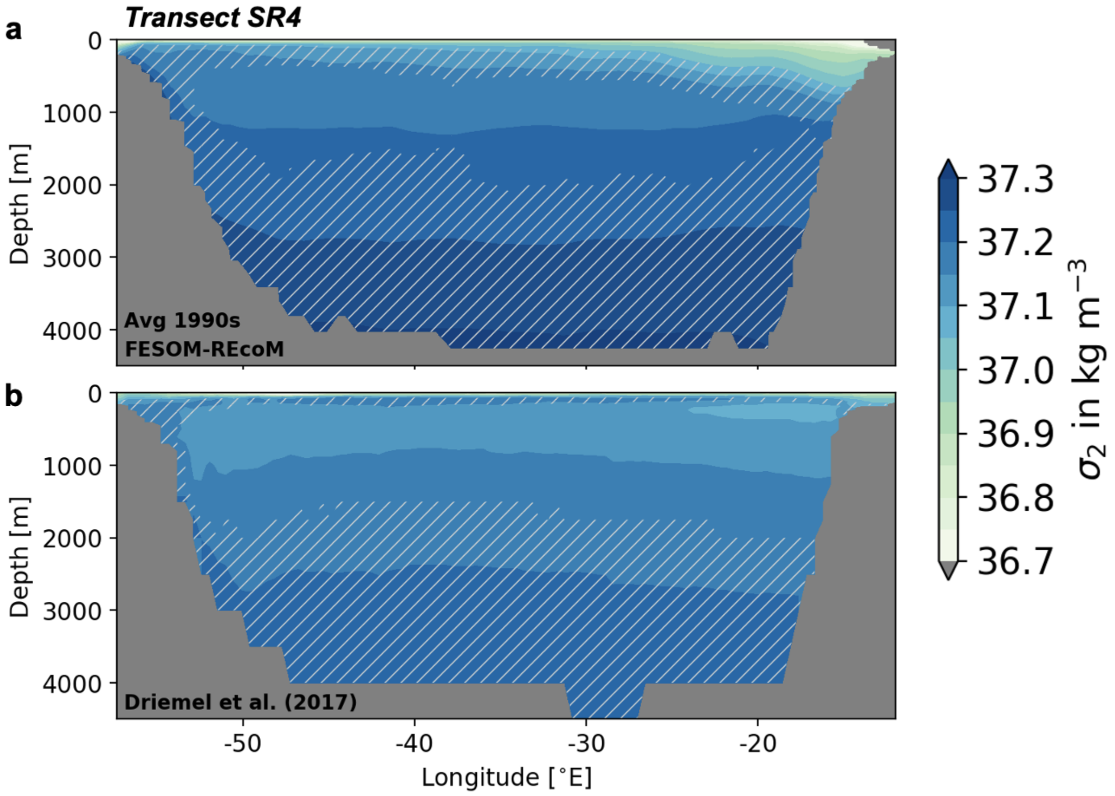

Figure S17: Model evaluation of density in the southern Weddell Sea. Potential density anomalies (σ_2 in kg m^{-3}, i.e., potential density referenced to 2000 dbar minus 1000 kg m^{-3}) at the transect SR4 of the World Ocean Circulation Experiment **a** as simulated for the 1990s in *simA* (historical + SSP5-8.5 scenario; see Methods in the main text) and **b** averaged over all available ship-based observations[4] . See Fig. 1 in the main text for the exact location of the transect SR4. In both panels, hatching denotes the presence of Weddell Sea Deep Water or Weddell Sea Bottom Water, defined in the model as waters with a potential temperature $<$-0.2°C and a practical salinity$>$34.55. This definition differs slightly from that used for the observed distribution of these two water masses, for which a temperature threshold of 0°C and practical salinity thresholds of 34.6 (Weddell Sea Deep Water) and 34.63 (Weddell Sea Bottom Water), respectively, are used[5] . See also Method section of the main text.

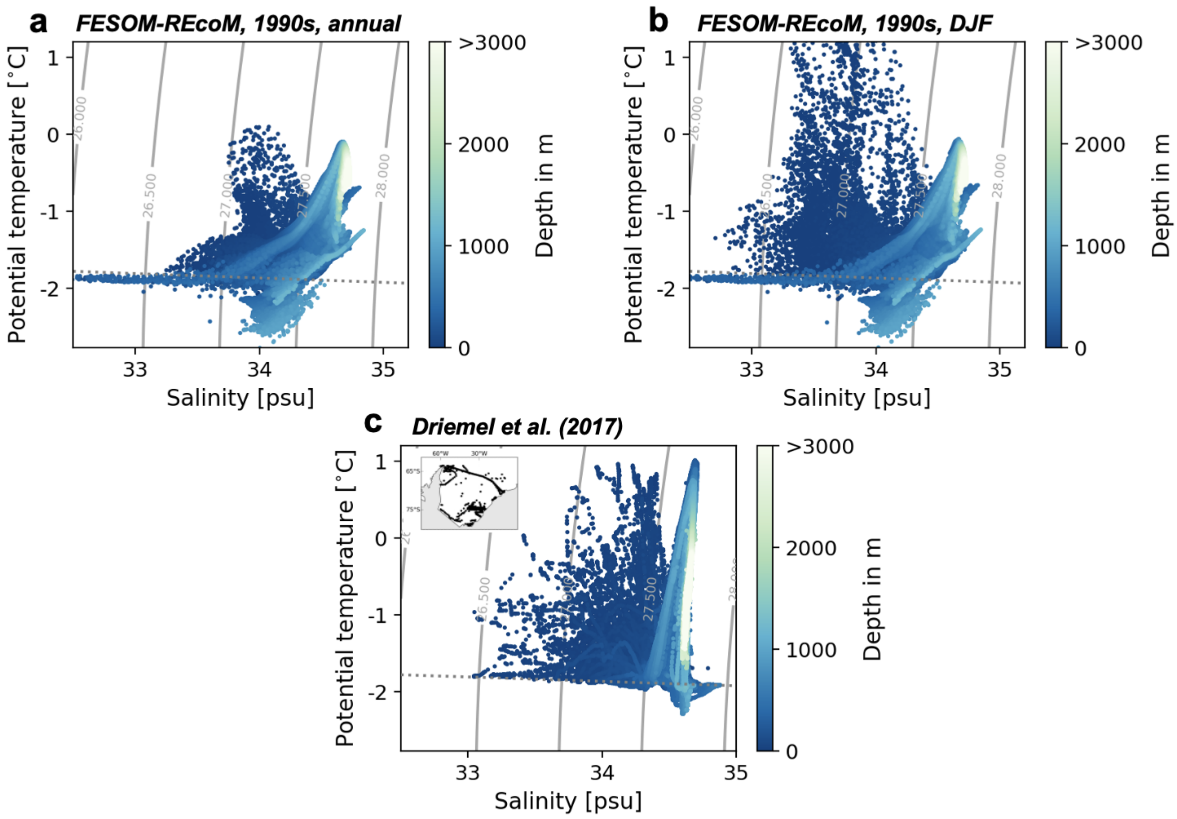

Figure S18: Model evaluation of temperature and salinity in the southern Weddell Sea. Temperature-Salinity diagrams as a function of the depth (see colors; m) of **a & b** FESOM-REcoM output for the area south of the transect SR4 in the 1990s in *simA* (historical + SSP5-8.5 scenario; see Methods in the main text) and **c** all available observations in the same area from Driemel et al.[4] . See map in inlet in panel **c** for the locations of the observations. For the model output, averages over **a** the whole year and **b** December, January, and February are shown, respectively, as 59% of all observations were taken in these three months. In all panels, grey contours in the background denote selected isopycnals of surface-referenced potential density.

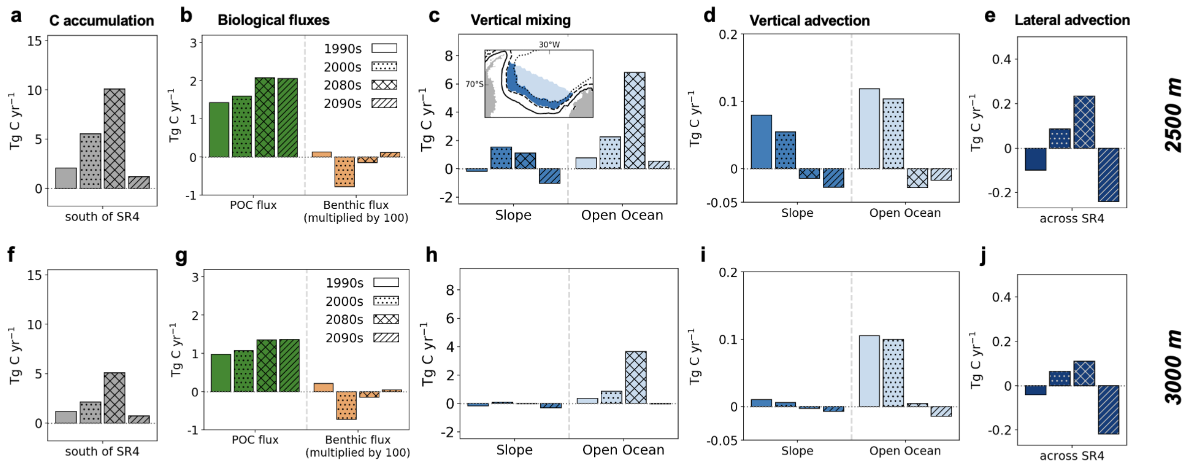

Figure S19: Southern Weddell Sea carbon budget below 2500 m and 3000 m. Average annual **a & f** total carbon accumulation rate, **b & g** sinking flux of particulate organic carbon (POC; green) and dissolved inorganic carbon fluxes from the sediments (orange), and divergence of physical flux components, namely **c & h** vertical mixing, **d & i** vertical advection, and **e & j** lateral advection in *simA* (historical + SSP5-8.5 scenario, see Methods in the main text). The contribution of other physical flux components is small and therefore not shown here (see Fig. 3 in the main text). All fluxes are given in Tg C yr^{-1} for the 1990s, 2000s, 2080s, and 2090s, as indicated by the hatching of the bars. Further, all fluxes are integrated over the area south of the transect SR4 of the World Ocean Circulation Experiment (see inlet in panel **c**) below **a-e** 2500 m and **f-j** 3000 m, respectively.

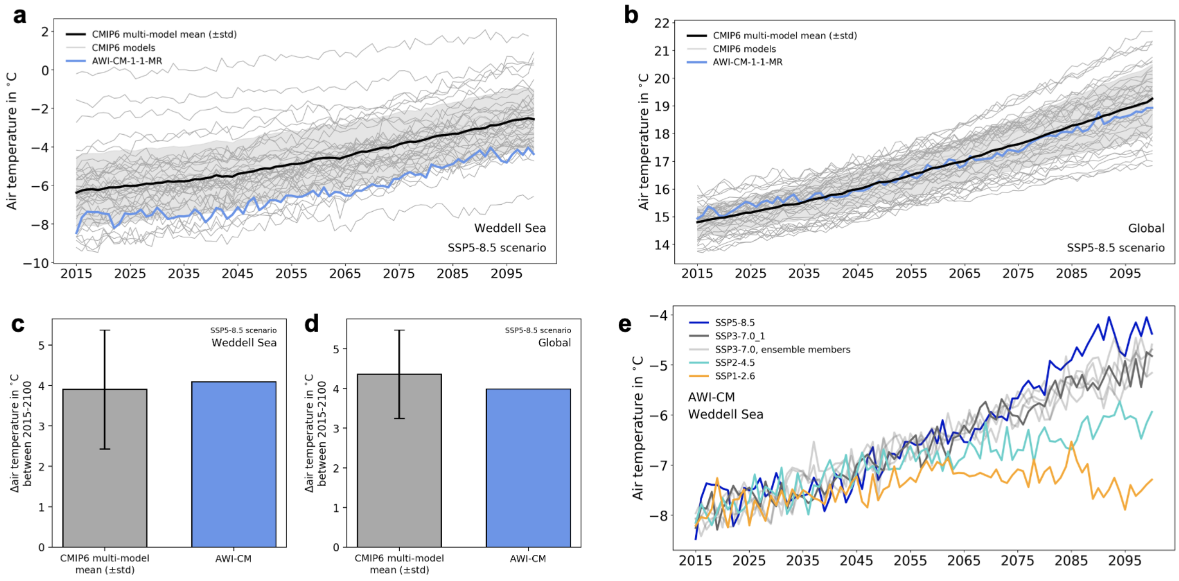

Figure S20: Air temperatures in the AWI Climate Model in comparison to other climate models. **a, b** Evolution of air temperatures in °C between 2015 and 2100 under the high-emission SSP5-8.5 scenario in 41 models contributing to the "Coupled Model Intercomparison Project Phase 6" (CMIP6) averaged **a** over the Weddell Sea and **b** globally. If for any climate model, multiple ensemble members are available for the SSP5-8.5 scenario in the CMIP6 archive, only the first ensemble member is shown here. In both panels, the thick black line shows the multi-model mean, the shading the multi-model mean ± one standard deviation, and the light blue line denotes the AWI Climate Model (AWI-CM), which is used to force the simulations in this study. The bars in panels **c, d** show the average warming between 2015 and 2100 of all CMIP6 models (grey; whisker denotes ± one standard deviation around the mean) and the AWI-CM (light blue) under the SSP5-8.5 scenario and averaged **c** over the Weddell Sea and **d** globally. Panel **e** shows the evolution of Weddell Sea air temperatures in the AWI-CM under a variety of emission scenarios. Note that multiple ensemble members of the AWI-CM are only available for the SSP3-7.0 scenario[6] . All CMIP6 data are available via the Earth System Grid Federation (https://esgf-data.dkrz.de/projects/cmip6-dkrz/, last access December 29, 2021).

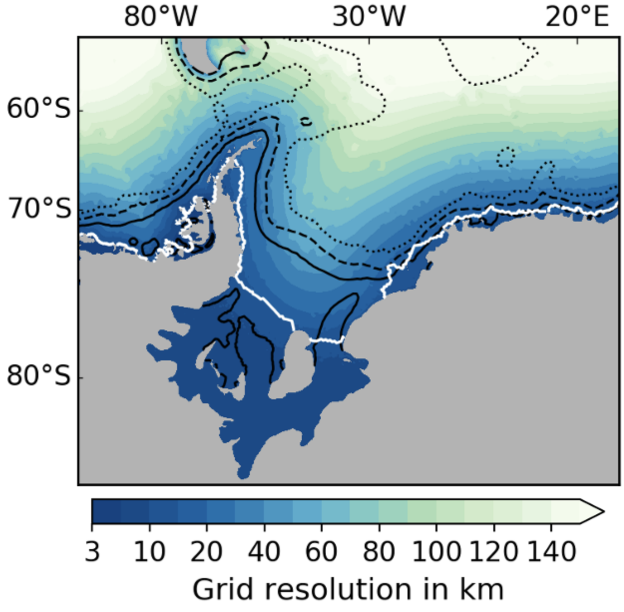

Figure S21: Horizontal grid resolution in the Weddell Sea sector of the global FESOM-REcoM simulations in this study. Black contours show the 700 m (solid), 2000 m (dashed), and 3500 m (dotted) isobaths, the white contour indicates the ice-shelf front.

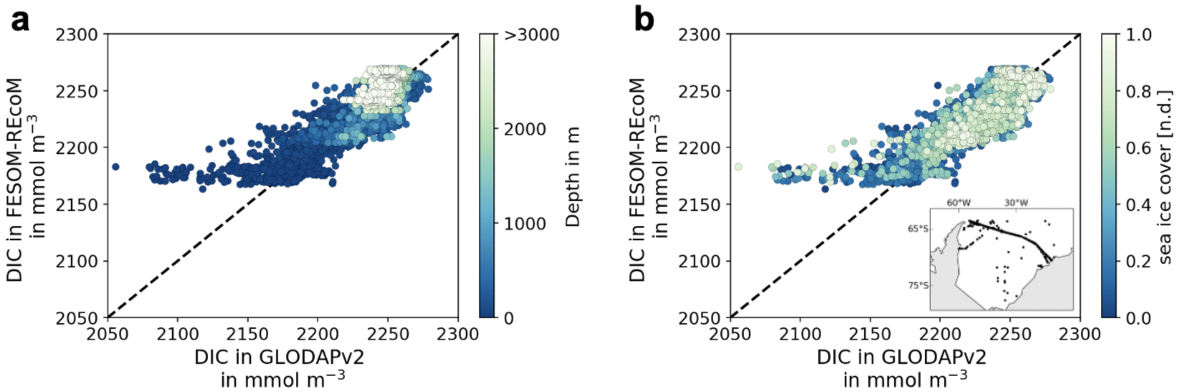

Figure S22: Model evaluation of dissolved inorganic carbon in the southern Weddell Sea. Scatter plot of observed (x-axis) and simulated (y-axis) concentrations of dissolved inorganic carbon (DIC; mmol C m^{-3}) in the southern Weddell Sea as a function of **a** the depth of the respective observation and **b** the simulated sea ice concentration (dimensionless). Simulated DIC concentrations are extracted from monthly model output of *simA* (historical + SSP5-8.5 scenario; see Methods in the main text) at the location and timing closest to that of the respective observation. The ship-based DIC observations are taken from GLODAPv2[7] , and the locations of all available observations in the area of interest for this study are given in the inlet in panel **b**.

Supplementary References

[1] Beaulieu, C. and Killick, R. “Distinguishing Trends and Shifts from Memory in Climate Data”. *Journal of Climate* **31** (2018), 9519–9543. DOI: 10.1175/JCLI-D-17-0863.1.

[2] Killick, R. et al. *EnvCpt: Detection of structural changes in climate and environment time series. R package version 1.1.3*. https://github.com/rkillick/EnvCpt/. https://cran.r-project.org/web/packages/EnvCpt/EnvCpt.pdf. 2021.

[3] Heuzé, C. et al. “Southern Ocean bottom water characteristics in CMIP5 models”. *Geophysical Research Letters* **40** (2013), 1409–1414. DOI: 10.1002/grl.50287.

[4] Driemel, A. et al. “From pole to pole: 33 years of physical oceanography onboard R/V Polarstern”. *Earth System Science Data* **9** (2017), 211–220. DOI: 10.5194/essd-9-211-2017.

[5] Fahrbach, E. et al. “Transport and structure of the Weddell Gyre”. *Ann. Geophys.* **12** (1994), 840–855. DOI: 10.1007/s00585-994-0840-7.

[6] Semmler, T. et al. “Simulations for CMIP6 With the AWI Climate Model AWI-CM-1-1”. *Journal of Advances in Modeling Earth Systems* **12** (2020), 1–34. DOI: 10.1029/2019MS002009.

[7] Olsen, A. et al. “The Global Ocean Data Analysis Project version 2 (GLODAPv2) – an internally consistent data product for the world ocean”. *Earth System Science Data* **8** (2016), 297–323. DOI: 10.5194/essd-8-297-2016.
